# Microgeographic population structuring of *Aedes aegypti* (Diptera: Culicidae)

**DOI:** 10.1371/journal.pone.0185150

**Published:** 2017-09-20

**Authors:** André Barretto Bruno Wilke, Ramon Wilk-da-Silva, Mauro Toledo Marrelli

**Affiliations:** Departamento de Epidemiologia, Faculdade de Saúde Pública, Universidade de São Paulo, São Paulo, Brasil; University of Malaya, MALAYSIA

## Abstract

*Aedes aegypti* is one of the species most favored by changes in the environment caused by urbanization. Its abundance increases rapidly in the face of such changes, increasing the risk of disease transmission. Previous studies have shown that mosquito species that have adapted to anthropogenic environmental changes benefit from urbanization and undergo population expansion. In light of this, we used microsatellite markers to explore how urbanization processes may be modulating *Ae*. *aegypti* populations collected from three areas with different levels of urbanization in the city of São Paulo, Brazil. Specimens were collected at eleven sites in three areas with different degrees of urbanization in the city of São Paulo: conserved, intermediate and urbanized. Ten microsatellite loci were used to characterize the populations from these areas genetically. Our findings suggest that as urbanized areas grow and the human population density in these areas increases, *Ae*. *aegypti* populations undergo a major population expansion, which can probably be attributed to the species’ adaptability to anthropogenic environmental changes. Our findings reveal a robust association between, on the one hand, urbanization processes and densification of the human population and, on the other, *Ae*. *aegypti* population structure patterns and population expansion. This indicates that this species benefits from anthropogenic effects, which are intensified by migration of the human population from rural to urban areas, increasing the risk of epidemics and disease transmission to an ever-increasing number of people.

## Background

Urbanization continues to increase as more and more people migrate from rural to urban areas. Indeed, there are now more people living in cities than in rural areas. This phenomenon is responsible for the extinction of species that are not adapted to the urban environment and is especially important for the epidemiology of vector-borne diseases, as there is a clear association between reduced species richness in urban areas and an increased incidence of these diseases [1,2]. This decrease in species richness can in turn be a result of human changes to the environment and the consequent increase in abundance of the few mosquito species that are adapted to the urbanized environment, very often with epidemiological consequences [2–4]. Mosquito surveys performed in urban parks in the metropolitan region of São Paulo, Brazil, indicated high abundance of species with epidemiological relevance, including the dengue vector, *Aedes aegypti* [5–7].

Dengue is a tropical disease caused by a flavivirus transmitted by *Aedes* mosquitoes. It endangers over 2 billion people of the world's population, causing approximately 390 million infections a year [8]. *Ae*. *aegypti* [9], which can be found in tropical and subtropical regions, is the main vector of dengue and also one of the mosquitoes that is best adapted to the urban environment; it can complete its entire life cycle within a human domicile, laying eggs in artificial breeding sites and blood feeding on human hosts [10].

For these reasons, *Ae*. *aegypti* is one of the most favored species when a rural area is urbanized. In this situation there is a rapid increase in its abundance and a consequent increase in the risk of dengue transmission [11–13]. The dynamics of dengue epidemics are fueled by several factors that are usually present in developing countries, such as unplanned urbanization, chaotic population growth and ineffective public health systems and vector surveillance [2,12,14,15].

Also, *Ae*. *aegypti* is considered the primary vector for the Zika and chikungunya viruses, as well as several other arboviruses, and is responsible for numerous outbreaks of insect-borne diseases worldwide [16,17]. Identifying the genetic structure of urban populations of *Ae*. *aegypti*, especially on a microgeographic scale, can lead to a better understanding of how human modifications to the physical environment are modulating population structure in this mosquito and the implications of this modulation for disease transmission and vector control.

Microsatellites are highly polymorphic neutral markers often used in genetic population studies [18]. Recently, their use has provided valuable information on microgeographic population structures of insects in urbanized areas [19–21]. Using DNA microsatellite loci, Multini et al. [19] found that *Aedes fluviatilis* populations in São Paulo, Brazil, had undergone population expansion primarily as a result of urban growth, highlighting the epidemiological significance of the association between urbanization processes and population structuring in vector mosquitoes.

In light of the high abundance of *Ae*. *aegypti* in the city of São Paulo and the major role played by this species in the transmission of the dengue, Zika and chikungunya viruses, this study used microsatellite markers to explore how urbanization processes can modulate populations of *Ae*. *aegypti* collected in three areas with different levels of urbanization in the city of São Paulo.

## Methods

### Specimen collection

*Ae*. *aegypti* mosquitoes were collected from eleven sites, no more than 30 km apart, in three areas with different levels of urbanization in the city of São Paulo, Brazil.

**Conserved areas** (CON): five municipal parks consisting of large green areas (with more than 97% of vegetal cover) open to the public from 5:00 am to 8:00 pm. No biological or chemical measures are used to control mosquitoes in the parks, which are home to wild birds, reptiles and mammals. **Intermediate areas** (INT): four collection sites on the University of São Paulo Armando de Salles Oliveira campus (with 80% of vegetal cover). The campus extends over 3,648,944.40 m^2^, of which some 800,000 m^2^ has been built on. Over 100,000 people travel through, visit or work or study on the campus every day. **Urbanized areas** (URB): two collection sites on the University of São Paulo health sciences campus, which is in a highly urbanized, densely populated area extending over 83,050.82 m^2^ (with less than 3% of vegetation cover). Much of this area (79.923,72 m^2^) has been built on (Table 1).

**Table 1:** *Aedes aegypti* collection sites and collection data.

Collection Site	Code	Coordinates	N*	Collection Year
Anhanguera Park	CON-1	23°24′54”S, 46°47′60” W	13	2013
Eucalipto Park	CON-2	23°36’54“S, 46°45’18”W	30	2012
Independência Park	CON-3	26°35’60”S, 46°36’18”W	26	2015
Piqueri Park	CON-4	23°31’30“S, 46°35’30”W	30	2013
Previdência Park	CON-5	23°35’60“S, 46° 43’30”W	30	2015
University of São Paulo Student Accommodations	INT-1	23°33’18”S, 46°43’30”W	30	2014
School of Communication and Arts	INT-2	23°33’18“S, 46°43’30”W	29	2014
Physics Institute	INT-3	23°33’54“S, 46°44’60” W	30	2014
Veterinary School	INT-4	23°33’54”S, 46°44’60”W	29	2014
School of Public Health	URB-1	23°33’18”S, 46°40’30”W	30	2015
Medical School	URB-2	23°33’18”S, 46°40’30”W	30	2015

*Number of females used

Mosquito collections were performed monthly from 2012 to 2015. Adult mosquitoes were collected outdoors with portable battery-powered aspirators [22], and immature mosquitoes were collected with dippers and randomly selected for further analysis to avoid testing siblings. Immature specimens were kept under laboratory conditions and fed with fish food (Tetra BettaMin) until they developed into adults. Specimens were identified with the aid of taxonomic keys [23] and stored at -20°C until DNA was extracted.

The study was approved by the University of São Paulo Research Ethics Committee, and collection permits were provided by the City of São Paulo Department of the Environment and Green Areas.

### DNA extraction and polymerase chain reaction

DNA extractions were performed with the DNEasy Blood and Tissue Kit (Qiagen, Hilden, Germany) following the manufacturer’s instructions. Polymerase chain reactions (PCRs) were carried out individually with ten microsatellite primers originally designed by Chambers et al. [24] and Slotman et al. [25] (Table 2) using the SuperMix PCR kit (Invitrogen, Carlsbad, CA, USA).

**Table 2:** Microsatellite loci amplified in *Aedes aegypti*.

Locus	Sequences 5’-3’	Repetitive motif	T (°C)	Size range (bp)	References
A10	F: AATCGTGACGCGTCTTTTG	CT	60	232–242*(232–239)	Chambers et al. [24]
	R: TAACTGCATCGAGGGAAACC				
B07	F: CAAACAACGAACTGCTCACG	GA	60	100–272*(157–183)	Chambers et al. [24]
	R: TCGCAATTTCAACAGGTAGG				
AT1	F: CGTCGACGTTATCTCCTTGTT	AT	55	134–170*156–174)	Slotman et al. [25]
	R: GGACCGGAAAGACACAGACA				
AG7	F: CGTGCGAGTGAATGAGAGAC	GA	55	112–190*(153–185)	Slotman et al. [25]
	R: CATCCTCTCATCAGCTTCTAATAAA				
AG2	F: TCCCCTTTCAAACCTAATGG	AG	55	96–152*(115–178)	Slotman et al. [25]
	R: TTTGCCCTCGTATGCTCTCT				
AG5	F: TGATCTTGAGAAGGCATCCA	AG	55	140–164*(170–180)	Slotman et al. [25]
	R: CGTTATCCTTTCATCACTTGTTTG				
AC7	F: TCGGCAAATTACCACAAACA	CA	55	106–130*(129–143)	Slotman et al. [25]
	R: CATTGGACTCGCTATAACACACA				
AC5	F: TGGATTGTTCTTAACAAACACGAT	CA	55	104–156*(149–163)	Slotman et al. [25]
	R: CGATCTCACTACGGGTTTCG				
AC1	F: TCCGGTGGGTTAAGGATAGA	CA	55	140–196*(193–209)	Slotman et al. [25]
	R: ACTTCACGCTCCAGCAATCT				
AG1	F: AATCCCCACACAAACACACC	AG	55	90–106*(113–129)	Slotman et al. [25]
	R: GGCCGTGGTGTTACTCTCTC				

T = annealing temperature, bp = base pairs

*Values found in the *Aedes aegypti* tested. In parentheses, size range found in the original study.

Ten microsatellite primers were labeled with a fluorescent dye (FAM, HEX or NED) (Thermo Fisher Scientific, Waltham, MA, USA), and the PCRs were carried out according to the manufacture’s protocol using an E6331000025 Eppendorf Thermocycler (Mastercycler Nexus Gradient, Eppendorf, Hamburg, Germany). PCR products were visualized on a 1% agarose gel stained with GelRed™ (Biotium, Hayward, CA, USA).

PCR products were diluted 1:7 by mixing 3 μL of each product labeled with a different dye with 21 μL of Ultra-Pure Water (Applied Biosystems, Foster City, CA, USA) to a final volume of 30 μL. Next, 2 μL of the diluted PCR products were mixed with 8.925 μL of Hi-Di formamide (Applied Biosystems, Foster City, CA, USA) and 0.075 μL of the size standard GeneScan-500 ROX (Applied Biosystems, Foster City, CA, USA) to a final volume of 11 μL. The samples were submitted to the University of São Paulo Center for Human Genome Studies and size-sorted in an ABI 3730 automatic sequencer (Applied Biosystems, Foster City, CA, USA). Fragments were sized with GeneMarker (v1.85 SoftGenetics, State College, PA, USA).

### Genetic analysis

Allele frequency, observed heterozygosity (H_O_) and expected heterozygosity (H_E_), deviations from the Hardy-Weinberg equilibrium, inbreeding coefficient (F_IS_) and pairwise F_ST_ with significance values (after 10,000 permutations) were calculated with Arlequin (v3.5) [26]. Linkage disequilibrium (using Bonferroni-corrected *P*-values) and number of migrants (Nm) were calculated using Genepop (v4.2 http://genepop.curtin.edu.au/) [27]. Allelic richness and private allelic richness were calculated using HP-Rare (v1.0) [28].

The probability of null alleles, genetic heterogeneity (F_ST_) and Cavalli-Sforza and Edwards chord distance (taking into account the null allele bias) were calculated per locus per population with FreeNa [29]. A Mantel test was used to compare both F_ST_ values using Past (v2.17c) [30].

A dendrogram based on the Cavalli-Sforza and Edwards chord distance was constructed using Statistica (v7.0) [31]. A linear correlation analyses between F_ST_/(1-F_ST_) and geographic distance (km) was performed using Past (v2.17c).

Bayesian analysis was performed with Structure (v2.3.3) [32], and the number of clusters (K) representing the best fit for the data was calculated with Structure Harvester (Web v0.6.94) [33]. Bottleneck (v1.2.2) was used to test for heterozygosity deficiency using the Stepwise Mutation Model (SMM) [34–36], a signature of population expansion (H_E_<H_EQ_), or heterozygosity excess, a signature of a bottleneck event (H_E_>H_EQ_), where H_E_ = the expected heterozygosity based on allele frequencies and H_EQ_ = the expected heterozygosity based on observed alleles.

## Results

### Marker assessment

Linkage disequilibrium for the 495 possible tests, per locus per population, was found only between loci A10 and AC7, AG2 and AC5, AT1 and B07, AT1 and AG2 and AT1 and AG7 after Bonferroni correction (*P =* 0.0001). These results are not considered significant because there were no repetitions of pairs of loci combinations across the populations tested.

The estimate for the probability of null alleles was high (>0.2) for locus AC1 in five populations, AC5 in three, AC7 in two, AG1 in seven, AG2 in two, AG5 in one, AG7 in one, AT1 in two and B07 in one. Locus A10 had a low probability of null alleles (<0.2) (S1 Table). Allelic richness ranged from 4.8 (CON-1) to 6.38 (CON-4), and private allelic richness from 0.13 (CON-1) to 0.43 (CON-5) (S2 Table). Hardy-Weinberg equilibrium tests were performed for each locus and population. H_E_ was higher than H_O_ in 103 of 110 possible tests, and average F_IS_ was 0.35 (S3 Table).

### Genetic differentiation

The results for F_ST_ indicated significant genetic structure between populations. Values ranged from 0.00629 to 0.11169, and only 7% of the values (4 out of 55) were not significant. When the potential bias due to null alleles was taken into account, the results ranged from 0.002133 to 0.093649. There were no statistically significant differences between the corrected and uncorrected F_ST_ values (r = 0.986, *P* <0.0001) (Table 3).

**Table 3:** Pairwise F_ST_* estimates for *Aedes aegypti* populations.

	CON-1	CON-2	CON-3	CON-4	CON-5	INT-1	INT-2	INT-3	INT-4	URB-1	URB-2
CON-1	-	0.03702	0.01002	0.02572	0.02288	0.04623	0.03654	0.05234	0.03347	0.03411	0.08131
CON-2	**0.05607**	-	0.02190	0.02702	0.03122	0.04340	0.06356	0.09364	0.03765	0.05332	0.07913
CON-3	0.02546	**0.02873**	-	0.00505	0.00837	0.02641	0.01798	0.04967	0.01815	0.02624	0.06727
CON-4	**0.03912**	**0.03013**	0.00629	-	0.00213	0.02540	0.01845	0.03103	0.01027	0.03539	0.05266
CON-5	**0.04003**	**0.03813**	0.01320	0.00663	-	0.02117	0.01910	0.03939	0.01902	0.02437	0.03992
INT-1	**0.06617**	**0.05217**	**0.03149**	**0.02723**	**0.02787**	-	0.03339	0.06738	0.05008	0.05172	0.07275
INT-2	**0.05498**	**0.08431**	**0.02893**	**0.02648**	**0.02762**	**0.04627**	-	0.04515	0.02662	0.03921	0.06233
INT-3	**0.07058**	**0.11169**	**0.05802**	**0.04074**	**0.05214**	**0.08097**	**0.05280**	-	0.05777	0.06322	0.08800
INT-4	**0.04804**	**0.05050**	**0.02227**	**0.01768**	**0.02562**	**0.06004**	**0.03408**	**0.06863**	-	0.03269	0.05180
URB-1	**0.04879**	**0.07165**	**0.03582**	**0.04371**	**0.03371**	**0.06853**	**0.05312**	**0.07553**	**0.03861**	-	0.06039
URB-2	**0.10940**	**0.10668**	**0.09181**	**0.07164**	**0.05299**	**0.09418**	**0.07653**	**0.11107**	**0.06932**	**0.07741**	-

*Below the diagonal: F_ST_ values without correction for null alleles. Significant values are in bold.

Above the diagonal: FreeNA corrected F_ST_ values.

Number of migrants between the populations was 3.94766 per population per generation based on 30 specimens per population, showing a low degree of allelic similarity. There was no correlation between genetic and geographical distances (r = 0,18; r^2^ = 0,03; *P* = 0,18) between populations.

### Genetic distance

**Fig 1:**
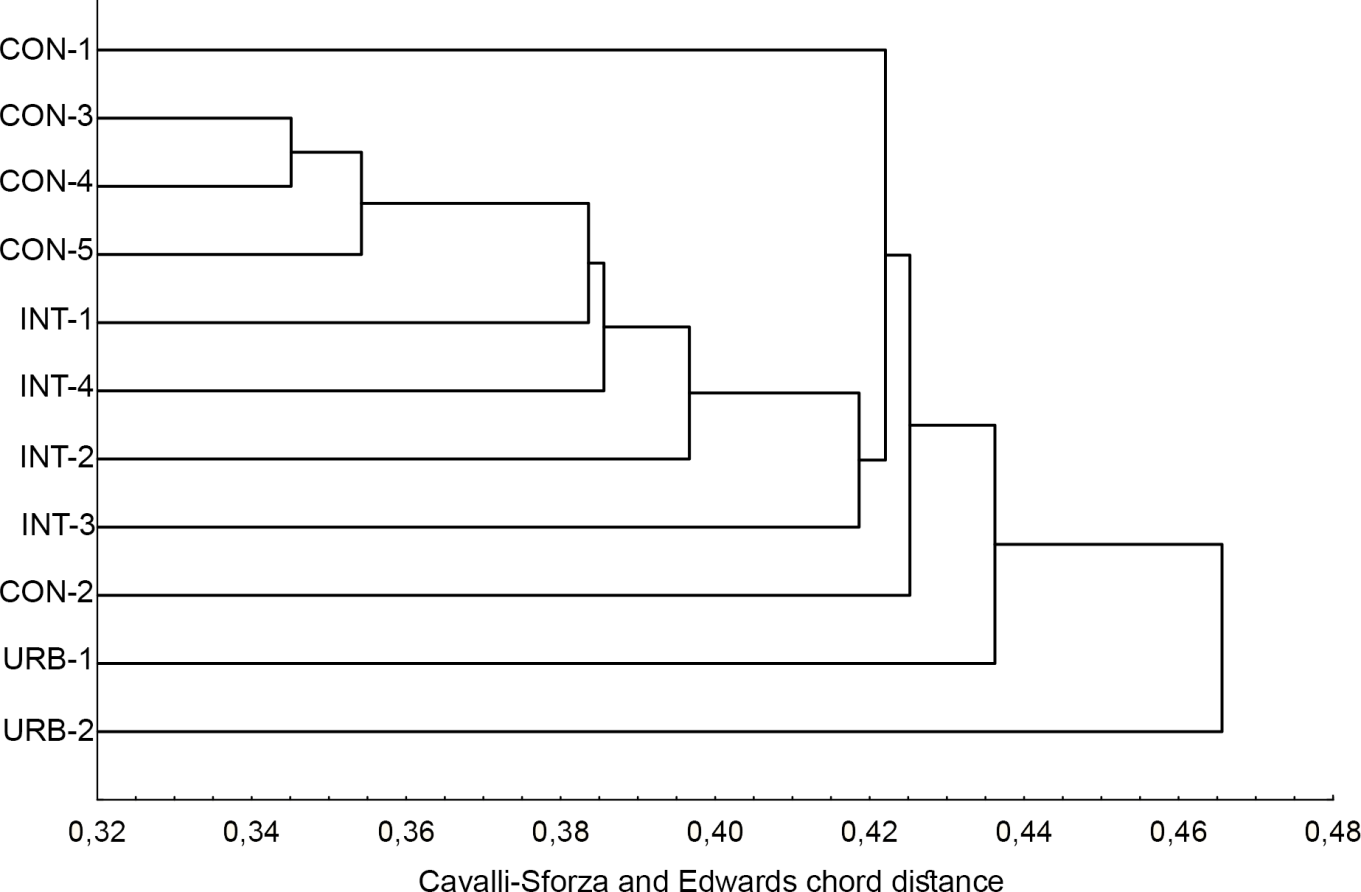
Genetic-distance dendrogram for *Aedes aegypti* based on Cavalli-Sforza and Edwards chord distance.

The dendrogram based on Cavalli-Sforza and Edwards chord distance was consistent with the level of urbanization in the areas where the specimens were collected. All eleven populations from the three urban areas grouped separately with no overlapping between populations from different areas. The two populations from highly urbanized areas, URB-1 and URB-2, were the most distinct, followed by two from the conserved area (CON-2 and CON-1, in that order). The CON-3, CON-4 and CON-5 populations segregated close to each other, as did the INT-1, INT-2, INT-3 and INT-4 populations (Fig 1).

### Bayesian cluster analysis

**Fig 2:**
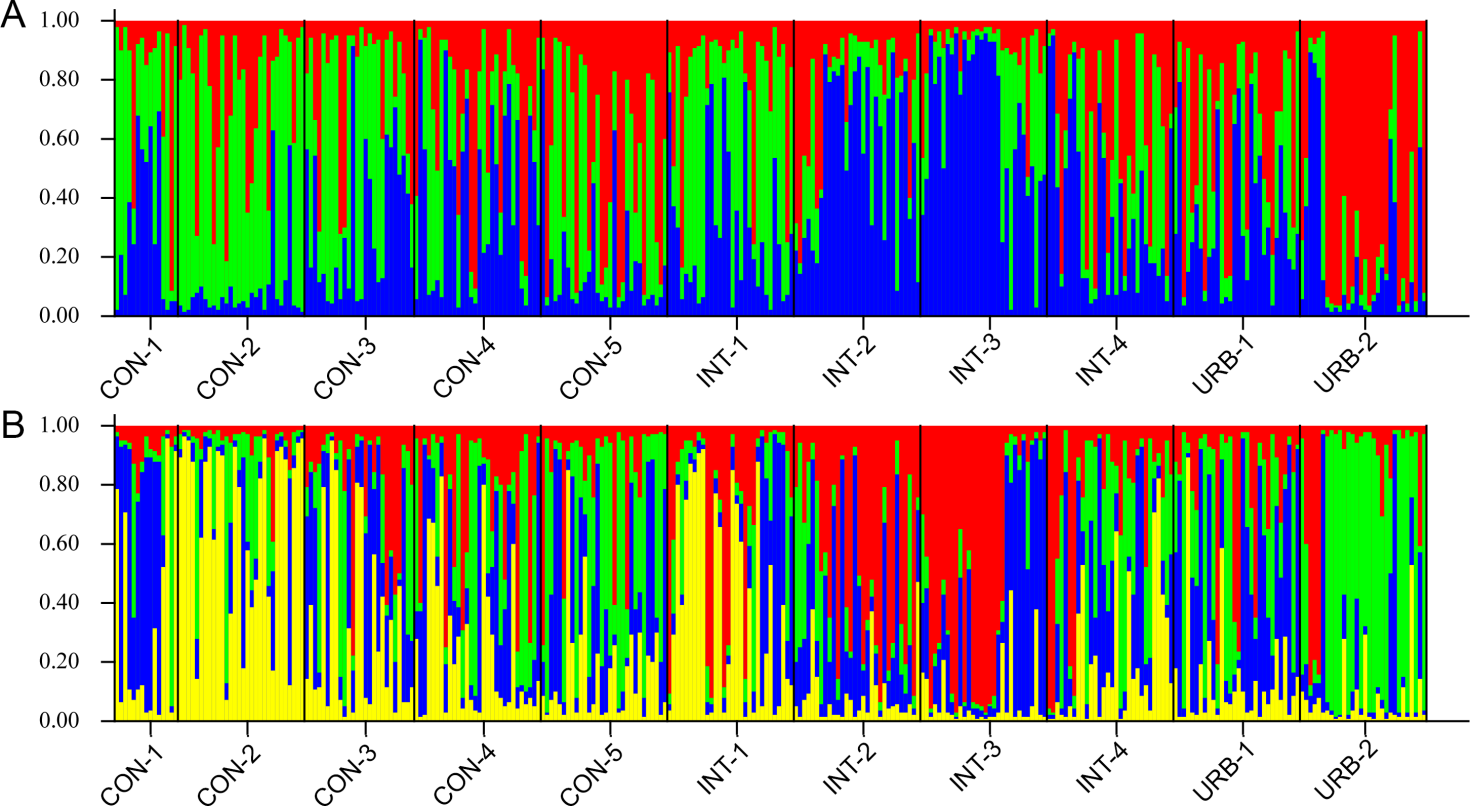
Bayesian analysis of population structure for all *Aedes aegypti* populations showing the subdivision of individuals for K = 3 (A) and K = 4 (B). Each of the 311 individuals from eleven populations is represented by a vertical line divided into different colored segments. The length of each segment represents the probability of the individual belonging to the genetic cluster represented by that color.

The method described by Evanno et al. [33] was used with the results of the Bayesian analysis to identify the K value that best represents the number of genetic groups in the populations tested. The K value found in this way was 4 (S1 Fig). Two subsequent analyses with different K values were performed. K = 3 indicated that the segregation of the populations depended on the area where the specimens were collected. For the CON populations, the color green predominated; for the INT populations, blue; and for the URB populations, red (Fig 2A). K = 4 showed that the URB-2 population belongs to a different genetic group than the remaining populations; this group is represented by the color yellow. The CON and INT populations formed a distinct genetic cluster in which green and red, respectively, predominated (Fig 2B).

### Population expansion analysis

Tests to assess heterozygosity deficiency using the SMM showed that there was a significant degree of deficiency in 5 of the 11 populations tested (*P*<0.05). Although not observed in any of the CON populations, heterozygosity deficiency was detected in all the INT and URB populations (except for INT-4), indicating a recent population expansion (Table 4) in the more urbanized areas. In a further analysis in which all 310 specimens were considered a metapopulation, all 10 loci displayed heterozygosity deficiency (H_E_<H_EQ_) with a highly significant *P*-value (0.00015). This result supports the hypothesis that the *Ae*. *aegypti* populations in this study have suffered a major recent population expansion.

**Table 4:** Tests to identify heterozygosity deficiency in *Aedes aegypti*.

		CON-1	CON-2	CON-3	CON-4	CON-5	INT-1	INT-2	INT-3	INT-4	URB-1	URB-2
**SMM**	H_E_ < H_EQ_	4	5	5	7	6	8	8	8	6	8	8
	H_E_ > H_EQ_	6	5	5	3	4	2	2	2	4	2	2
	*P* (H_E_ < H_EQ_)	0.61024	0.36864	0.39316	0.06188	0.17758	**0.01291**	**0.01421**	**0.01439**	0.18442	**0.01456**	**0.01583**

Number of loci with heterozygosity excess (H_E_) and number of loci with expected heterozygosity excess based on the number of observed alleles (H_EQ_) under the SMM. Significant *P*-values (<0.05) for heterozygosity deficit in bold.

## Discussion

The global distribution of *Ae*. *aegypti* is intimately connected with man-made changes to the physical environment. While urbanization modifies the ecosystem to create an environment that is more suitable for one particular species, *Homo sapiens* [1], the mosquito *Ae*. *aegypti* also benefits from these changes. This situation, allied to the lack of sanitation and sewage treatment and inadequate epidemiologic surveillance common in developing countries, results in ideal conditions for a major increase in the abundance of this species, increasing the risk of disease transmission.

Our findings suggest that the *Ae*. *aegypti* populations in this study have undergone a major population expansion, probably as a result of increased urbanization and human population density as well as the species’ adaptability to anthropogenic changes in the physical environment. When urbanized areas expand and human population density increases, the availability of human hosts and breeding sites increases, while larval competition and the number of natural predators decrease. This agrees with the findings of Multini et al. [19] for *Ae*. *fluviatilis*, Donnelly et al. [36] for *Anopheles arabiensis* and *Anopheles gambiae*, Michel [37] for *Anopheles funestus* and Mirabello & Conn [38] for *Anopheles darlingi*.

The *Ae*. *aegypti* populations from more preserved ecosystems (CON) did not show signs of population expansion, unlike the INT and URB populations, corroborating the hypothesis that the urban environment benefits this species and that it is highly adapted to human changes to the landscape.

Bayesian analysis showed that structuring has been occurring in *Ae*. *aegypti* populations and is correlated with the level of urbanization, supported by the lack of correlation between genetic and geographic distances. This can be explained by the fact that this species is well adapted to the urban environment and therefore does not have to actively seek new areas in order to find hosts or breeding sites as these are widely available in cities regardless of the level of urbanization and are plentiful even in the more conserved environments, such as urban parks.

A possible hypothesis to explain this situation is that after *Ae*. *aegypti* was reintroduced in Brazil in the 1970’s [39], the growth of the city favored its expansion, preventing the genetic structuring of its populations. However, once urbanization in a given area was complete and these populations reached their peak abundance, genetic structuring restarted, resulting in the population structures observed here. This hypothesis is corroborated by the low Nm between populations despite their geographic proximity as revealed by the dendrogram in Fig 1 and the Bayesian analysis.

Further, the domiciliation of *Ae*. *aegypti*, which resulted from anthropogenic impacts on its evolution may be closely associated with microevolutionary processes [40], such as the structuring found in this study. The results for allelic and private allelic richness for *Ae*. *aegypti* were consistent with the results of previous studies of this species [20,39,41], indicating that the loci used here revealed the genuine allelic richness for the populations in this study.

Our finding of urbanization-dependent structuring is of great significance for the dynamics of disease transmission, particularly because there appears to be low Nm between the populations collected in areas with different levels of urbanization in this study. Furthermore, with increasing urbanization the demographic expansion of *Ae*. *aegypti* populations tends to intensify since this species is favored by urbanization. As a result, there is an increase in diseases transmission indirectly caused by human effects on the environment, such as the development of urban heat islands [42], and a consequent increase in the incidence of dengue [13].

The findings of this study indicate a robust association between, on the one hand, urbanization and increasing human population density and, on the other, *Ae*. *aegypti* population structure patterns and population expansion. This suggests that *Ae*. *aegypti* benefits from human changes to the environment, which increase in intensity as the human population migrates from rural to urban areas, increasing the risk of disease transmission and epidemics for an ever-increasing number of people.

## Supporting information

S1 Fig Graph of ΔK against K showing K = 4 as the most probable number of genetic clusters. (TIF)

S1 Table Null allele frequency estimates per locus per *Aedes aegypti* population. (DOCX)

S2 Table Allele frequencies for the ten loci analyzed in *Aedes aegypti* populations. Allelic richness (*Na*) and private allelic richness (*Np*).
(DOCX)

S3 Table Characterization of microsatellite loci in *Aedes aegypti*. Significant *P*-values in bold.
(DOCX)
