# Supplementary figures and images for: Microgeographic population structuring of Aedes aegypti (Diptera: Culicidae)
Source: PLoS One. 2017 Sep 20;12(9):e0185150. doi: 10.1371/journal.pone.0185150 (PMC5607186; doi:10.1371/journal.pone.0185150)

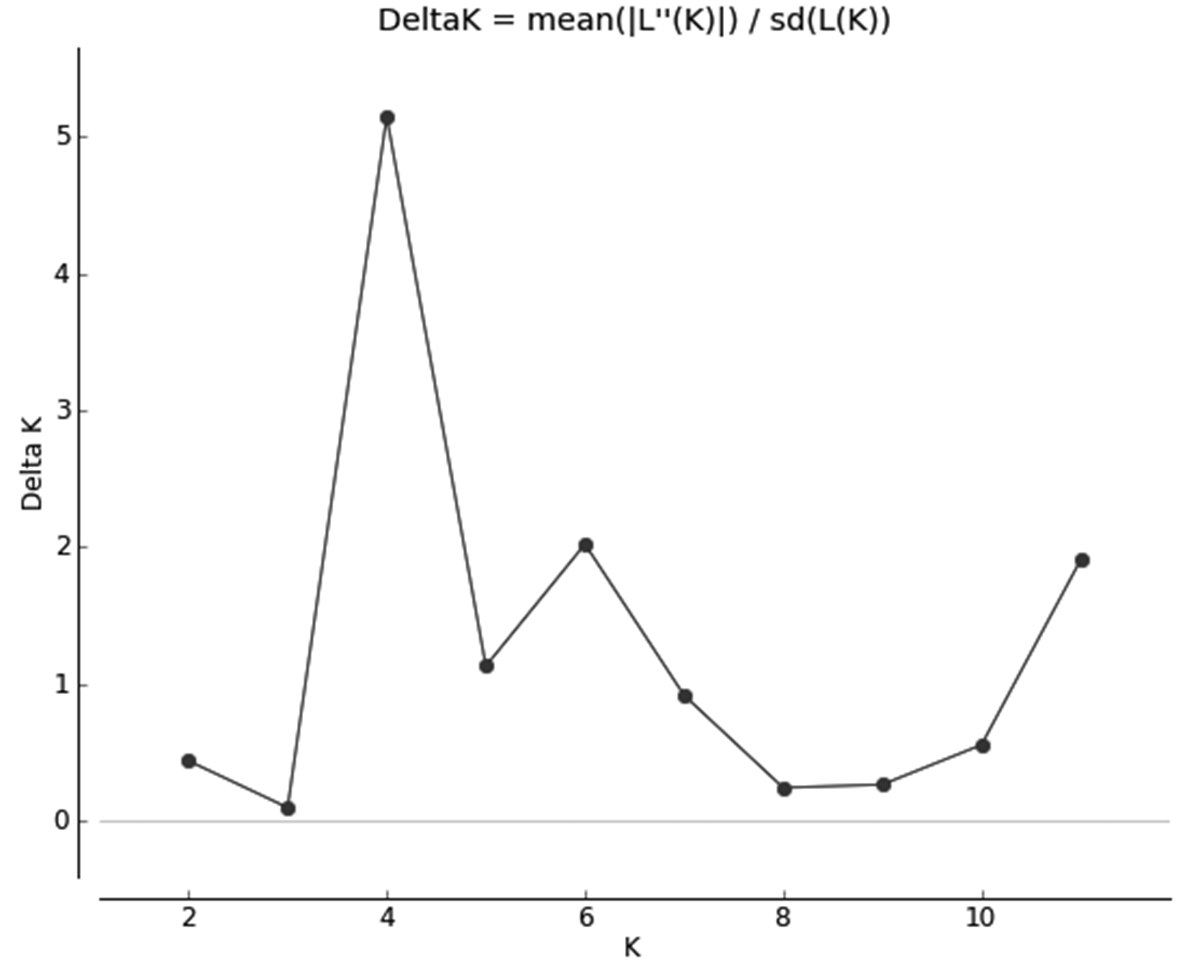

Supplement: S1 Fig — (TIF) [file pone.0185150.s001.tif]
