# Supplementary material for: Microgeographic population structuring of Aedes aegypti (Diptera: Culicidae)
Source: PLoS One. 2017 Sep 20;12(9):e0185150. doi: 10.1371/journal.pone.0185150 (PMC5607186; doi:10.1371/journal.pone.0185150)
Supplement: S1 Table — (DOCX) [file pone.0185150.s002.docx]

**S1 table.** Null allele frequency estimates per locus per *Aedes aegypti* population*.*

| Locus | Population | Estimate of null allele frequency |  | Locus | Population | Estimate of null allele frequency |
| --- | --- | --- | --- | --- | --- | --- |
| AT1 | CON- 1 | 0.19276 |  | AT1 | INT-2 | 0.04672 |
| AG7 |  | 0.05778 |  | AG7 |  | 0.05007 |
| AC1 |  | 0.20001 |  | AC1 |  | 0.10850 |
| AG2 |  | 0.25897 |  | AG2 |  | 0.20790 |
| AG5 |  | 0.14705 |  | AG5 |  | 0.15062 |
| AC5 |  | 0.04445 |  | AC5 |  | 0.11762 |
| AG1 |  | 0.20411 |  | AG1 |  | 0.31448 |
| A10 |  | 0.09174 |  | A10 |  | 0.00000 |
| B07 |  | 0.18118 |  | B07 |  | 0.09523 |
| AC7 |  | 0.17030 |  | AC7 |  | 0.11996 |
| AT1 | CON-2 | 0.06452 |  | AT1 | INT-3 | 0.20416 |
| AG7 |  | 0.13178 |  | AG7 |  | 0.04545 |
| AC1 |  | 0.21418 |  | AC1 |  | 0.15103 |
| AG2 |  | 0.13574 |  | AG2 |  | 0.03920 |
| AG5 |  | 0.02337 |  | AG5 |  | 0.03340 |
| AC5 |  | 0.23738 |  | AC5 |  | 0.18328 |
| AG1 |  | 0.28445 |  | AG1 |  | 0.20134 |
| A10 |  | 0.01614 |  | A10 |  | 0.03382 |
| B07 |  | 0.00000 |  | B07 |  | 0.19041 |
| AC7 |  | 0.08025 |  | AC7 |  | 0.31651 |
| AT1 | CON-3 | 0.33658 |  | AT1 | INT-4 | 0.08485 |
| AG7 |  | 0.07501 |  | AG7 |  | 0.00000 |
| AC1 |  | 0.09228 |  | AC1 |  | 0.19002 |
| AG2 |  | 0.07744 |  | AG2 |  | 0.06010 |
| AG5 |  | 0.10099 |  | AG5 |  | 0.17451 |
| AC5 |  | 0.16066 |  | AC5 |  | 0.27383 |
| AG1 |  | 0.31333 |  | AG1 |  | 0.24886 |
| A10 |  | 0.15029 |  | A10 |  | 0.08435 |
| B07 |  | 0.12565 |  | B07 |  | 0.02688 |
| AC7 |  | 0.07593 |  | AC7 |  | 0.22674 |
| AT1 | CON-4 | 0.07490 |  | AT1 | URB-1 | 0.10469 |
| AG7 |  | 0.01369 |  | AG7 |  | 0.30684 |
| AC1 |  | 0.11119 |  | AC1 |  | 0.21962 |
| AG2 |  | 0.16744 |  | AG2 |  | 0.04047 |
| AG5 |  | 0.15895 |  | AG5 |  | 0.22139 |
| AC5 |  | 0.24106 |  | AC5 |  | 0.12293 |
| AG1 |  | 0.08262 |  | AG1 |  | 0.11803 |
| A10 |  | 0.02769 |  | A10 |  | 0.00013 |
| B07 |  | 0.25327 |  | B07 |  | 0.08014 |
| AC7 |  | 0.10510 |  | AC7 |  | 0.19314 |
| AT1 | CON-5 | 0.11183 |  | AT1 | URB-2 | 0.19952 |
| AG7 |  | 0.02016 |  | AG7 |  | 0.01699 |
| AC1 |  | 0.28519 |  | AC1 |  | 0.19730 |
| AG2 |  | 0.13330 |  | AG2 |  | 0.00000 |
| AG5 |  | 0.10920 |  | AG5 |  | 0.00001 |
| AC5 |  | 0.15508 |  | AC5 |  | 0.14978 |
| AG1 |  | 0.03935 |  | AG1 |  | 0.37452 |
| A10 |  | 0.12479 |  | A10 |  | 0.11951 |
| B07 |  | 0.14976 |  | B07 |  | 0.18369 |
| AC7 |  | 0.11123 |  | AC7 |  | 0.08471 |
| AT1 | INT-1 | 0.00000 |  |  |  |  |
| AG7 |  | 0.15697 |  |  |  |  |
| AC1 |  | 0.20940 |  |  |  |  |
| AG2 |  | 0.04993 |  |  |  |  |
| AG5 |  | 0.05334 |  |  |  |  |
| AC5 |  | 0.17601 |  |  |  |  |
| AG1 |  | 0.16911 |  |  |  |  |
| A10 |  | 0.18470 |  |  |  |  |
| B07 |  | 0.09956 |  |  |  |  |
| AC7 |  | 0.06806 |  |  |  |  |
