# Supplementary material for: Microgeographic population structuring of Aedes aegypti (Diptera: Culicidae)
Source: PLoS One. 2017 Sep 20;12(9):e0185150. doi: 10.1371/journal.pone.0185150 (PMC5607186; doi:10.1371/journal.pone.0185150)
Supplement: S2 Table — Allelic richness (Na) and private allelic richness (Np). (DOCX) [file pone.0185150.s003.docx]

**S2 Table.** Allele frequencies for the ten loci analyzed in *Aedes aegypti* populations.

| Locus | Allele | CON-1 | CON-2 | CON-3 | CON-4 | CON-5 | INT-1 | INT-2 | INT-3 | INT-4 | URB-1 | URB-2 |
| --- | --- | --- | --- | --- | --- | --- | --- | --- | --- | --- | --- | --- |
| Locus: AT1 | 134 | 0.000 | 0.017 | 0.000 | 0.000 | 0.000 | 0.000 | 0.000 | 0.000 | 0.000 | 0.000 | 0.000 |
|  | 138 | 0.000 | 0.000 | 0.000 | 0.000 | 0.000 | 0.000 | 0.017 | 0.000 | 0.017 | 0.000 | 0.000 |
|  | 140 | 0.300 | 0.317 | 0.212 | 0.267 | 0.317 | 0.467 | 0.167 | 0.217 | 0.217 | 0.400 | 0.300 |
|  | 142 | 0.333 | 0.333 | 0.115 | 0.067 | 0.167 | 0.217 | 0.200 | 0.100 | 0.250 | 0.183 | 0.133 |
|  | 144 | 0.000 | 0.033 | 0.212 | 0.150 | 0.200 | 0.067 | 0.200 | 0.350 | 0.167 | 0.217 | 0.183 |
|  | 146 | 0.000 | 0.000 | 0.077 | 0.083 | 0.033 | 0.050 | 0.183 | 0.100 | 0.017 | 0.033 | 0.083 |
|  | 148 | 0.167 | 0.017 | 0.038 | 0.000 | 0.000 | 0.000 | 0.000 | 0.017 | 0.000 | 0.000 | 0.050 |
|  | 150 | 0.133 | 0.117 | 0.154 | 0.167 | 0.167 | 0.017 | 0.050 | 0.017 | 0.233 | 0.017 | 0.050 |
|  | 152 | 0.000 | 0.000 | 0.000 | 0.000 | 0.033 | 0.017 | 0.000 | 0.000 | 0.000 | 0.017 | 0.000 |
|  | 154 | 0.000 | 0.050 | 0.096 | 0.100 | 0.050 | 0.133 | 0.067 | 0.000 | 0.017 | 0.000 | 0.033 |
|  | 156 | 0.000 | 0.000 | 0.000 | 0.033 | 0.033 | 0.017 | 0.017 | 0.017 | 0.033 | 0.000 | 0.117 |
|  | 158 | 0.000 | 0.117 | 0.038 | 0.033 | 0.000 | 0.000 | 0.017 | 0.000 | 0.000 | 0.050 | 0.000 |
|  | 160 | 0.000 | 0.000 | 0.038 | 0.033 | 0.000 | 0.000 | 0.000 | 0.033 | 0.017 | 0.017 | 0.000 |
|  | 166 | 0.033 | 0.000 | 0.019 | 0.033 | 0.000 | 0.017 | 0.033 | 0.117 | 0.033 | 0.000 | 0.017 |
|  | 170 | 0.033 | 0.000 | 0.000 | 0.033 | 0.000 | 0.000 | 0.050 | 0.033 | 0.000 | 0.067 | 0.033 |
|  |  |  |  |  |  |  |  |  |  |  |  |  |
|  |  | CON-1 | CON-2 | CON-3 | CON-4 | CON-5 | INT-1 | INT-2 | INT-3 | INT-4 | URB-1 | URB-2 |
| Locus: AG7 | 112 | 0.000 | 0.000 | 0.000 | 0.000 | 0.000 | 0.000 | 0.017 | 0.000 | 0.000 | 0.000 | 0.000 |
|  | 124 | 0.000 | 0.000 | 0.000 | 0.000 | 0.000 | 0.000 | 0.000 | 0.017 | 0.000 | 0.000 | 0.000 |
|  | 136 | 0.133 | 0.050 | 0.038 | 0.083 | 0.050 | 0.133 | 0.200 | 0.067 | 0.083 | 0.000 | 0.083 |
|  | 138 | 0.000 | 0.000 | 0.000 | 0.000 | 0.017 | 0.000 | 0.000 | 0.000 | 0.000 | 0.000 | 0.000 |
|  | 140 | 0.233 | 0.467 | 0.308 | 0.517 | 0.333 | 0.450 | 0.433 | 0.417 | 0.450 | 0.233 | 0.283 |
|  | 142 | 0.167 | 0.150 | 0.135 | 0.067 | 0.150 | 0.033 | 0.067 | 0.067 | 0.050 | 0.000 | 0.133 |
|  | 144 | 0.000 | 0.050 | 0.077 | 0.017 | 0.050 | 0.017 | 0.017 | 0.083 | 0.017 | 0.000 | 0.017 |
|  | 146 | 0.033 | 0.033 | 0.058 | 0.067 | 0.033 | 0.100 | 0.050 | 0.017 | 0.050 | 0.000 | 0.067 |
|  | 148 | 0.000 | 0.000 | 0.019 | 0.000 | 0.017 | 0.133 | 0.067 | 0.000 | 0.000 | 0.000 | 0.000 |
|  | 150 | 0.400 | 0.250 | 0.308 | 0.233 | 0.333 | 0.133 | 0.133 | 0.333 | 0.333 | 0.733 | 0.367 |
|  | 152 | 0.000 | 0.000 | 0.019 | 0.000 | 0.000 | 0.000 | 0.000 | 0.000 | 0.000 | 0.000 | 0.000 |
|  | 160 | 0.000 | 0.000 | 0.000 | 0.000 | 0.000 | 0.000 | 0.000 | 0.000 | 0.000 | 0.033 | 0.000 |
|  | 180 | 0.033 | 0.000 | 0.000 | 0.000 | 0.000 | 0.000 | 0.000 | 0.000 | 0.000 | 0.000 | 0.000 |
|  | 186 | 0.000 | 0.000 | 0.038 | 0.017 | 0.000 | 0.000 | 0.017 | 0.000 | 0.017 | 0.000 | 0.000 |
|  | 190 | 0.000 | 0.000 | 0.000 | 0.000 | 0.017 | 0.000 | 0.000 | 0.000 | 0.000 | 0.000 | 0.050 |
|  |  |  |  |  |  |  |  |  |  |  |  |  |
|  |  | CON-1 | CON-2 | CON-3 | CON-4 | CON-5 | INT-1 | INT-2 | INT-3 | INT-4 | URB-1 | URB-2 |
| Locus: AC1 | 140 | 0.000 | 0.000 | 0.000 | 0.000 | 0.033 | 0.000 | 0.017 | 0.000 | 0.000 | 0.033 | 0.000 |
|  | 144 | 0.000 | 0.000 | 0.000 | 0.000 | 0.033 | 0.000 | 0.000 | 0.000 | 0.000 | 0.000 | 0.000 |
|  | 150 | 0.000 | 0.000 | 0.000 | 0.000 | 0.100 | 0.000 | 0.000 | 0.000 | 0.000 | 0.000 | 0.000 |
|  | 178 | 0.000 | 0.000 | 0.000 | 0.000 | 0.000 | 0.000 | 0.000 | 0.000 | 0.000 | 0.033 | 0.000 |
|  | 180 | 0.033 | 0.000 | 0.077 | 0.100 | 0.067 | 0.167 | 0.167 | 0.200 | 0.067 | 0.050 | 0.083 |
|  | 184 | 0.267 | 0.150 | 0.288 | 0.133 | 0.050 | 0.117 | 0.200 | 0.083 | 0.183 | 0.067 | 0.033 |
|  | 190 | 0.000 | 0.000 | 0.000 | 0.000 | 0.033 | 0.000 | 0.000 | 0.000 | 0.000 | 0.033 | 0.000 |
|  | 192 | 0.300 | 0.267 | 0.192 | 0.183 | 0.217 | 0.383 | 0.217 | 0.267 | 0.150 | 0.317 | 0.300 |
|  | 194 | 0.400 | 0.583 | 0.442 | 0.583 | 0.467 | 0.333 | 0.400 | 0.433 | 0.600 | 0.467 | 0.583 |
|  | 196 | 0.000 | 0.000 | 0.000 | 0.000 | 0.000 | 0.000 | 0.000 | 0.017 | 0.000 | 0.000 | 0.000 |
|  |  |  |  |  |  |  |  |  |  |  |  |  |
|  |  | CON-1 | CON-2 | CON-3 | CON-4 | CON-5 | INT-1 | INT-2 | INT-3 | INT-4 | URB-1 | URB-2 |
| Locus: AG2 | 96 | 0.000 | 0.000 | 0.000 | 0.000 | 0.000 | 0.000 | 0.000 | 0.000 | 0.000 | 0.000 | 0.017 |
|  | 98 | 0.000 | 0.000 | 0.000 | 0.000 | 0.000 | 0.000 | 0.000 | 0.000 | 0.000 | 0.000 | 0.017 |
|  | 100 | 0.000 | 0.050 | 0.192 | 0.117 | 0.117 | 0.200 | 0.200 | 0.017 | 0.100 | 0.233 | 0.217 |
|  | 102 | 0.067 | 0.100 | 0.019 | 0.100 | 0.017 | 0.067 | 0.067 | 0.133 | 0.133 | 0.067 | 0.033 |
|  | 104 | 0.633 | 0.200 | 0.308 | 0.333 | 0.217 | 0.350 | 0.217 | 0.600 | 0.067 | 0.267 | 0.250 |
|  | 114 | 0.000 | 0.000 | 0.000 | 0.000 | 0.000 | 0.000 | 0.000 | 0.000 | 0.050 | 0.000 | 0.000 |
|  | 116 | 0.000 | 0.000 | 0.038 | 0.000 | 0.000 | 0.000 | 0.000 | 0.000 | 0.083 | 0.033 | 0.000 |
|  | 118 | 0.000 | 0.000 | 0.000 | 0.017 | 0.183 | 0.000 | 0.050 | 0.000 | 0.000 | 0.000 | 0.000 |
|  | 120 | 0.000 | 0.000 | 0.038 | 0.017 | 0.000 | 0.000 | 0.000 | 0.033 | 0.083 | 0.000 | 0.017 |
|  | 122 | 0.000 | 0.000 | 0.019 | 0.000 | 0.067 | 0.000 | 0.000 | 0.000 | 0.017 | 0.017 | 0.000 |
|  | 124 | 0.000 | 0.000 | 0.077 | 0.033 | 0.083 | 0.067 | 0.117 | 0.050 | 0.067 | 0.083 | 0.067 |
|  | 126 | 0.000 | 0.000 | 0.019 | 0.017 | 0.000 | 0.000 | 0.050 | 0.000 | 0.000 | 0.017 | 0.000 |
|  | 128 | 0.000 | 0.033 | 0.038 | 0.033 | 0.183 | 0.100 | 0.217 | 0.017 | 0.000 | 0.067 | 0.017 |
|  | 130 | 0.067 | 0.000 | 0.000 | 0.050 | 0.000 | 0.000 | 0.017 | 0.017 | 0.017 | 0.000 | 0.000 |
|  | 132 | 0.000 | 0.000 | 0.019 | 0.000 | 0.000 | 0.000 | 0.000 | 0.017 | 0.000 | 0.000 | 0.000 |
|  | 134 | 0.000 | 0.000 | 0.000 | 0.067 | 0.000 | 0.000 | 0.000 | 0.067 | 0.000 | 0.000 | 0.000 |
|  | 136 | 0.000 | 0.117 | 0.000 | 0.050 | 0.000 | 0.000 | 0.000 | 0.000 | 0.050 | 0.000 | 0.033 |
|  | 138 | 0.067 | 0.067 | 0.019 | 0.050 | 0.050 | 0.000 | 0.000 | 0.000 | 0.167 | 0.067 | 0.067 |
|  | 140 | 0.100 | 0.367 | 0.096 | 0.067 | 0.067 | 0.133 | 0.017 | 0.033 | 0.083 | 0.150 | 0.200 |
|  | 142 | 0.067 | 0.050 | 0.058 | 0.050 | 0.017 | 0.067 | 0.050 | 0.000 | 0.083 | 0.000 | 0.067 |
|  | 144 | 0.000 | 0.017 | 0.019 | 0.000 | 0.000 | 0.000 | 0.000 | 0.017 | 0.000 | 0.000 | 0.000 |
|  | 150 | 0.000 | 0.000 | 0.000 | 0.000 | 0.000 | 0.017 | 0.000 | 0.000 | 0.000 | 0.000 | 0.000 |
|  | 152 | 0.000 | 0.000 | 0.038 | 0.000 | 0.000 | 0.000 | 0.000 | 0.000 | 0.000 | 0.000 | 0.000 |
|  |  |  |  |  |  |  |  |  |  |  |  |  |
|  |  | CON-1 | CON-2 | CON-3 | CON-4 | CON-5 | INT-1 | INT-2 | INT-3 | INT-4 | URB-1 | URB-2 |
| Locus: AG5 | 140 | 0.000 | 0.033 | 0.000 | 0.000 | 0.000 | 0.000 | 0.000 | 0.000 | 0.000 | 0.000 | 0.000 |
|  | 150 | 0.133 | 0.067 | 0.077 | 0.083 | 0.000 | 0.000 | 0.100 | 0.083 | 0.083 | 0.000 | 0.017 |
|  | 152 | 0.200 | 0.167 | 0.346 | 0.300 | 0.283 | 0.583 | 0.533 | 0.267 | 0.350 | 0.317 | 0.267 |
|  | 154 | 0.033 | 0.167 | 0.000 | 0.083 | 0.183 | 0.083 | 0.050 | 0.017 | 0.117 | 0.100 | 0.200 |
|  | 156 | 0.067 | 0.017 | 0.038 | 0.033 | 0.067 | 0.117 | 0.067 | 0.050 | 0.000 | 0.000 | 0.033 |
|  | 158 | 0.000 | 0.017 | 0.077 | 0.033 | 0.100 | 0.000 | 0.000 | 0.000 | 0.000 | 0.050 | 0.150 |
|  | 160 | 0.133 | 0.083 | 0.192 | 0.183 | 0.150 | 0.067 | 0.150 | 0.400 | 0.117 | 0.117 | 0.067 |
|  | 162 | 0.333 | 0.267 | 0.192 | 0.183 | 0.150 | 0.133 | 0.100 | 0.183 | 0.317 | 0.333 | 0.183 |
|  | 164 | 0.100 | 0.183 | 0.077 | 0.100 | 0.067 | 0.017 | 0.000 | 0.000 | 0.017 | 0.083 | 0.083 |
|  |  |  |  |  |  |  |  |  |  |  |  |  |
|  |  | CON-1 | CON-2 | CON-3 | CON-4 | CON-5 | INT-1 | INT-2 | INT-3 | INT-4 | URB-1 | URB-2 |
| Locus: AC5 | 104 | 0.000 | 0.000 | 0.000 | 0.000 | 0.000 | 0.000 | 0.000 | 0.000 | 0.000 | 0.000 | 0.017 |
|  | 112 | 0.000 | 0.000 | 0.000 | 0.000 | 0.000 | 0.017 | 0.000 | 0.000 | 0.000 | 0.000 | 0.000 |
|  | 130 | 0.067 | 0.033 | 0.077 | 0.033 | 0.117 | 0.117 | 0.117 | 0.100 | 0.017 | 0.133 | 0.083 |
|  | 138 | 0.000 | 0.000 | 0.000 | 0.000 | 0.000 | 0.000 | 0.000 | 0.000 | 0.083 | 0.000 | 0.000 |
|  | 140 | 0.467 | 0.133 | 0.212 | 0.200 | 0.167 | 0.150 | 0.200 | 0.183 | 0.267 | 0.200 | 0.050 |
|  | 142 | 0.467 | 0.800 | 0.692 | 0.733 | 0.617 | 0.667 | 0.600 | 0.717 | 0.533 | 0.633 | 0.283 |
|  | 144 | 0.000 | 0.033 | 0.019 | 0.033 | 0.100 | 0.050 | 0.083 | 0.000 | 0.100 | 0.033 | 0.483 |
|  | 152 | 0.000 | 0.000 | 0.000 | 0.000 | 0.000 | 0.000 | 0.000 | 0.000 | 0.000 | 0.000 | 0.017 |
|  | 154 | 0.000 | 0.000 | 0.000 | 0.000 | 0.000 | 0.000 | 0.000 | 0.000 | 0.000 | 0.000 | 0.050 |
|  | 156 | 0.000 | 0.000 | 0.000 | 0.000 | 0.000 | 0.000 | 0.000 | 0.000 | 0.000 | 0.000 | 0.017 |
|  |  |  |  |  |  |  |  |  |  |  |  |  |
|  |  | CON-1 | CON-2 | CON-3 | CON-4 | CON-5 | INT-1 | INT-2 | INT-3 | INT-4 | URB-1 | URB-2 |
| Locus: AG1 | 90 | 0.000 | 0.000 | 0.058 | 0.000 | 0.000 | 0.000 | 0.000 | 0.000 | 0.000 | 0.000 | 0.000 |
|  | 96 | 0.000 | 0.000 | 0.000 | 0.000 | 0.000 | 0.000 | 0.000 | 0.000 | 0.050 | 0.000 | 0.000 |
|  | 98 | 0.000 | 0.000 | 0.000 | 0.050 | 0.017 | 0.033 | 0.000 | 0.017 | 0.050 | 0.000 | 0.117 |
|  | 100 | 0.233 | 0.017 | 0.000 | 0.150 | 0.150 | 0.017 | 0.367 | 0.117 | 0.167 | 0.383 | 0.417 |
|  | 101 | 0.000 | 0.000 | 0.000 | 0.000 | 0.017 | 0.000 | 0.000 | 0.000 | 0.000 | 0.000 | 0.000 |
|  | 102 | 0.300 | 0.167 | 0.250 | 0.283 | 0.283 | 0.250 | 0.400 | 0.750 | 0.350 | 0.217 | 0.200 |
|  | 104 | 0.200 | 0.433 | 0.423 | 0.300 | 0.250 | 0.283 | 0.233 | 0.117 | 0.383 | 0.367 | 0.267 |
|  | 106 | 0.267 | 0.383 | 0.269 | 0.217 | 0.283 | 0.417 | 0.000 | 0.000 | 0.000 | 0.033 | 0.000 |
|  |  |  |  |  |  |  |  |  |  |  |  |  |
|  |  | CON-1 | CON-2 | CON-3 | CON-4 | CON-5 | INT-1 | INT-2 | INT-3 | INT-4 | URB-1 | URB-2 |
| Locus: A10 | 232 | 0.267 | 0.083 | 0.250 | 0.317 | 0.300 | 0.117 | 0.433 | 0.350 | 0.267 | 0.267 | 0.400 |
|  | 233 | 0.000 | 0.000 | 0.000 | 0.000 | 0.000 | 0.000 | 0.017 | 0.000 | 0.000 | 0.000 | 0.000 |
|  | 234 | 0.033 | 0.000 | 0.077 | 0.000 | 0.050 | 0.067 | 0.067 | 0.000 | 0.000 | 0.083 | 0.100 |
|  | 236 | 0.200 | 0.167 | 0.096 | 0.083 | 0.150 | 0.033 | 0.100 | 0.150 | 0.117 | 0.017 | 0.033 |
|  | 238 | 0.433 | 0.467 | 0.481 | 0.550 | 0.500 | 0.667 | 0.367 | 0.500 | 0.617 | 0.583 | 0.417 |
|  | 240 | 0.067 | 0.267 | 0.096 | 0.050 | 0.000 | 0.117 | 0.017 | 0.000 | 0.000 | 0.050 | 0.050 |
|  | 242 | 0.000 | 0.017 | 0.000 | 0.000 | 0.000 | 0.000 | 0.000 | 0.000 | 0.000 | 0.000 | 0.000 |
|  |  |  |  |  |  |  |  |  |  |  |  |  |
|  |  | CON-1 | CON-2 | CON-3 | CON-4 | CON-5 | INT-1 | INT-2 | INT-3 | INT-4 | URB-1 | URB-2 |
| Locus: B07 | 100 | 0.000 | 0.000 | 0.000 | 0.017 | 0.000 | 0.000 | 0.000 | 0.000 | 0.000 | 0.000 | 0.000 |
|  | 102 | 0.100 | 0.000 | 0.000 | 0.000 | 0.033 | 0.000 | 0.000 | 0.000 | 0.000 | 0.000 | 0.000 |
|  | 106 | 0.000 | 0.000 | 0.000 | 0.000 | 0.033 | 0.000 | 0.000 | 0.000 | 0.000 | 0.000 | 0.000 |
|  | 158 | 0.000 | 0.017 | 0.019 | 0.033 | 0.033 | 0.050 | 0.033 | 0.000 | 0.000 | 0.000 | 0.033 |
|  | 160 | 0.000 | 0.000 | 0.000 | 0.017 | 0.000 | 0.033 | 0.000 | 0.000 | 0.000 | 0.017 | 0.000 |
|  | 164 | 0.100 | 0.183 | 0.154 | 0.133 | 0.217 | 0.283 | 0.233 | 0.183 | 0.283 | 0.200 | 0.700 |
|  | 168 | 0.600 | 0.450 | 0.596 | 0.417 | 0.417 | 0.483 | 0.600 | 0.483 | 0.433 | 0.650 | 0.150 |
|  | 169 | 0.000 | 0.067 | 0.000 | 0.000 | 0.000 | 0.017 | 0.000 | 0.000 | 0.017 | 0.000 | 0.000 |
|  | 170 | 0.000 | 0.000 | 0.019 | 0.033 | 0.000 | 0.000 | 0.000 | 0.000 | 0.000 | 0.050 | 0.017 |
|  | 172 | 0.100 | 0.000 | 0.058 | 0.033 | 0.050 | 0.033 | 0.000 | 0.067 | 0.050 | 0.050 | 0.000 |
|  | 174 | 0.100 | 0.283 | 0.077 | 0.083 | 0.017 | 0.000 | 0.017 | 0.000 | 0.017 | 0.017 | 0.067 |
|  | 176 | 0.000 | 0.000 | 0.000 | 0.083 | 0.000 | 0.000 | 0.000 | 0.000 | 0.000 | 0.000 | 0.000 |
|  | 178 | 0.000 | 0.000 | 0.000 | 0.000 | 0.000 | 0.000 | 0.050 | 0.067 | 0.000 | 0.000 | 0.000 |
|  | 182 | 0.000 | 0.000 | 0.000 | 0.000 | 0.000 | 0.000 | 0.000 | 0.000 | 0.050 | 0.000 | 0.000 |
|  | 184 | 0.000 | 0.000 | 0.077 | 0.150 | 0.067 | 0.100 | 0.033 | 0.133 | 0.150 | 0.000 | 0.033 |
|  | 186 | 0.000 | 0.000 | 0.000 | 0.000 | 0.000 | 0.000 | 0.033 | 0.017 | 0.000 | 0.017 | 0.000 |
|  | 272 | 0.000 | 0.000 | 0.000 | 0.000 | 0.133 | 0.000 | 0.000 | 0.050 | 0.000 | 0.000 | 0.000 |
|  |  |  |  |  |  |  |  |  |  |  |  |  |
|  |  | CON-1 | CON-2 | CON-3 | CON-4 | CON-5 | INT-1 | INT-2 | INT-3 | INT-4 | URB-1 | URB-2 |
| Locus: AC7 | 106 | 0.000 | 0.000 | 0.038 | 0.000 | 0.000 | 0.000 | 0.000 | 0.200 | 0.100 | 0.100 | 0.100 |
|  | 108 | 0.000 | 0.033 | 0.000 | 0.000 | 0.000 | 0.000 | 0.000 | 0.167 | 0.000 | 0.033 | 0.033 |
|  | 110 | 0.000 | 0.000 | 0.000 | 0.000 | 0.000 | 0.000 | 0.000 | 0.000 | 0.000 | 0.033 | 0.000 |
|  | 112 | 0.933 | 0.783 | 0.846 | 0.650 | 0.633 | 0.583 | 0.750 | 0.483 | 0.850 | 0.717 | 0.467 |
|  | 114 | 0.067 | 0.000 | 0.000 | 0.033 | 0.050 | 0.183 | 0.100 | 0.033 | 0.000 | 0.000 | 0.017 |
|  | 118 | 0.000 | 0.000 | 0.000 | 0.000 | 0.000 | 0.000 | 0.017 | 0.000 | 0.000 | 0.000 | 0.033 |
|  | 122 | 0.000 | 0.000 | 0.000 | 0.000 | 0.000 | 0.000 | 0.000 | 0.017 | 0.000 | 0.050 | 0.000 |
|  | 124 | 0.000 | 0.000 | 0.000 | 0.017 | 0.067 | 0.033 | 0.050 | 0.083 | 0.033 | 0.050 | 0.033 |
|  | 126 | 0.000 | 0.183 | 0.115 | 0.150 | 0.183 | 0.133 | 0.033 | 0.017 | 0.017 | 0.017 | 0.250 |
|  | 127 | 0.000 | 0.000 | 0.000 | 0.033 | 0.000 | 0.000 | 0.017 | 0.000 | 0.000 | 0.000 | 0.000 |
|  | 128 | 0.000 | 0.000 | 0.000 | 0.117 | 0.067 | 0.067 | 0.000 | 0.000 | 0.000 | 0.000 | 0.017 |
|  | 130 | 0.000 | 0.000 | 0.000 | 0.000 | 0.000 | 0.000 | 0.033 | 0.000 | 0.000 | 0.000 | 0.050 |
|  |  |  |  |  |  |  |  |  |  |  |  |  |
| ***Na*** |  | 4.8 | 4.94 | 6.04 | 6.38 | 6.21 | 5.37 | 5.8 | 5.3 | 5.54 | 5.32 | 6.22 |
| ***Np*** |  | 0.13 | 0.21 | 0.29 | 0.2 | 0.43 | 0.13 | 0.2 | 0.16 | 0.4 | 0.32 | 0.44 |

Allelic richness (*Na*) and private allelic richness (*Np*).
