# Supplementary material for: Microgeographic population structuring of Aedes aegypti (Diptera: Culicidae)
Source: PLoS One. 2017 Sep 20;12(9):e0185150. doi: 10.1371/journal.pone.0185150 (PMC5607186; doi:10.1371/journal.pone.0185150)
Supplement: S3 Table — Significant P-values in bold. (DOCX) [file pone.0185150.s004.docx]

**S3 Table.** Characterization of microsatellite loci in *Aedes aegypti*

| **Population** | **Locus** | **Number of alleles** | **Observed Heterozygosity** | **Expected Heterozygosity** | ***Fis*** | ***P*** |
| --- | --- | --- | --- | --- | --- | --- |
| CON-1 | AT1 | 6 | 0.40000 | 0.77701 | 0.4940 | **0.00195** |
|  | AG7 | 6 | 0.66667 | 0.76322 | 0.1304 | 0.42983 |
|  | AC1 | 4 | 0.33333 | 0.70115 | 0.5333 | **0.00189** |
|  | AG2 | 6 | 0.20000 | 0.59080 | 0.6693 | **0.00024** |
|  | AG5 | 7 | 0.53333 | 0.82529 | 0.3618 | **0.02629** |
|  | AC5 | 3 | 0.46667 | 0.57931 | 0.2000 | 0.79757 |
|  | AG1 | 4 | 0.40000 | 0.77011 | 0.4894 | **0.00339** |
|  | A10 | 5 | 0.53333 | 0.71954 | 0.2656 | 0.06553 |
|  | B07 | 5 | 0.33333 | 0.62069 | 0.4717 | **0.01519** |
|  | AC7 | 2 | 0.00000 | 0.12874 | 1 | **0.03477** |
|  |  |  |  |  |  |  |
| CON-2 | AT1 | 8 | 0.66667 | 0.77006 | 0.1363 | **0.01711** |
|  | AG7 | 6 | 0.50000 | 0.70282 | 0.2921 | **0.00000** |
|  | AC1 | 3 | 0.23333 | 0.57571 | 0.5988 | **0.00000** |
|  | AG2 | 9 | 0.56667 | 0.80452 | 0.2992 | **0.00000** |
|  | AG5 | 9 | 0.83333 | 0.84068 | 0.0089 | **0.00000** |
|  | AC5 | 4 | 0.06667 | 0.34576 | 0.8098 | **0.00000** |
|  | AG1 | 4 | 0.16667 | 0.64802 | 0.7461 | **0.00000** |
|  | A10 | 5 | 0.66667 | 0.68757 | 0.0309 | **0.00351** |
|  | B07 | 5 | 0.80000 | 0.69040 | 0.1619 | **0.00049** |
|  | AC7 | 3 | 0.30000 | 0.35763 | 0.1635 | **0.02992** |
|  |  |  |  |  |  |  |
| CON-3 | AT1 | 10 | 0.23077 | 0.87029 | 0.7387 | **0.00000** |
|  | AG7 | 9 | 0.65385 | 0.79487 | 0.1803 | 0.54218 |
|  | AC1 | 4 | 0.53846 | 0.69155 | 0.2248 | **0.04130** |
|  | AG2 | 15 | 0.69231 | 0.85822 | 0.1964 | 0.12903 |
|  | AG5 | 7 | 0.61538 | 0.80241 | 0.2366 | **0.00802** |
|  | AC5 | 4 | 0.23077 | 0.47888 | 0.5231 | **0.00031** |
|  | AG1 | 4 | 0.15385 | 0.69608 | 0.7824 | **0.00000** |
|  | A10 | 5 | 0.46154 | 0.69532 | 0.3407 | **0.00008** |
|  | B07 | 7 | 0.42308 | 0.61689 | 0.3185 | 0.06528 |
|  | AC7 | 3 | 0.23077 | 0.27451 | 0.1620 | **0.03206** |
|  |  |  |  |  |  |  |
| CON-4 | AT1 | 11 | 0.70000 | 0.86610 | 0.1944 | **0.00000** |
|  | AG7 | 7 | 0.66667 | 0.67345 | 0.0102 | 0.10994 |
|  | AC1 | 4 | 0.40000 | 0.60847 | 0.3465 | **0.00065** |
|  | AG2 | 14 | 0.53333 | 0.85763 | 0.3822 | **0.00000** |
|  | AG5 | 8 | 0.53333 | 0.83051 | 0.3618 | **0.00000** |
|  | AC5 | 4 | 0.10000 | 0.42712 | 0.7689 | **0.00000** |
|  | AG1 | 5 | 0.60000 | 0.77062 | 0.2244 | **0.01581** |
|  | A10 | 4 | 0.56667 | 0.59774 | 0.0528 | 0.11174 |
|  | B07 | 10 | 0.33333 | 0.78136 | 0.5776 | **0.00000** |
|  | AC7 | 6 | 0.43333 | 0.54802 | 0.2121 | **0.04368** |
|  |  |  |  |  |  |  |
| CON-5 | AT1 | 8 | 0.56667 | 0.81186 | 0.3056 | **0.00022** |
|  | AG7 | 9 | 0.70000 | 0.76102 | 0.0814 | 0.17445 |
|  | AC1 | 8 | 0.23333 | 0.72712 | 0.6828 | **0.00000** |
|  | AG2 | 10 | 0.63333 | 0.86780 | 0.2736 | **0.00029** |
|  | AG5 | 7 | 0.63333 | 0.83616 | 0.2457 | **0.00000** |
|  | AC5 | 4 | 0.33333 | 0.57797 | 0.4274 | **0.00044** |
|  | AG1 | 6 | 0.70000 | 0.76667 | 0.0883 | 0.14119 |
|  | A10 | 4 | 0.50000 | 0.64576 | 0.2287 | **0.00005** |
|  | B07 | 9 | 0.53333 | 0.76384 | 0.3054 | **0.00095** |
|  | AC7 | 5 | 0.43333 | 0.56328 | 0.2337 | **0.01577** |
|  |  |  |  |  |  |  |
| INT-1 | AT1 | 9 | 0.70000 | 0.72147 | 0.0303 | 0.75243 |
|  | AG7 | 7 | 0.46667 | 0.74520 | 0.3778 | **0.00000** |
|  | AC1 | 4 | 0.33333 | 0.71243 | 0.5364 | **0.00000** |
|  | AG2 | 8 | 0.73333 | 0.80960 | 0.0957 | **0.00968** |
|  | AG5 | 6 | 0.60000 | 0.62712 | 0.0440 | 0.05320 |
|  | AC5 | 5 | 0.26667 | 0.52542 | 0.4967 | **0.00016** |
|  | AG1 | 5 | 0.40000 | 0.69379 | 0.4276 | **0.00000** |
|  | A10 | 5 | 0.23333 | 0.53164 | 0.5653 | **0.00000** |
|  | B07 | 7 | 0.50000 | 0.68249 | 0.2707 | **0.00075** |
|  | AC7 | 5 | 0.50000 | 0.61299 | 0.1869 | **0.00367** |
|  |  |  |  |  |  |  |
| INT-2 | AT1 | 11 | 0.76667 | 0.86158 | 0.1119 | 0.06134 |
|  | AG7 | 9 | 0.63333 | 0.75480 | 0.1632 | **0.02224** |
|  | AC1 | 5 | 0.53333 | 0.73729 | 0.2801 | **0.00115** |
|  | AG2 | 10 | 0.46667 | 0.85424 | 0.4579 | **0.00000** |
|  | AG5 | 6 | 0.43333 | 0.67740 | 0.3642 | **0.00005** |
|  | AC5 | 4 | 0.40000 | 0.58927 | 0.3249 | **0.00239** |
|  | AG1 | 3 | 0.13333 | 0.66215 | 0.8014 | **0.00000** |
|  | A10 | 6 | 0.96667 | 0.67401 | 0.4450 | **0.00000** |
|  | B07 | 7 | 0.43333 | 0.58927 | 0.2680 | 0.06049 |
|  | AC7 | 7 | 0.30000 | 0.42938 | 0.3049 | **0.03888** |
|  |  |  |  |  |  |  |
| INT-3 | AT1 | 10 | 0.43333 | 0.80734 | 0.4675 | **0.00000** |
|  | AG7 | 7 | 0.66667 | 0.71073 | 0.0630 | **0.02406** |
|  | AC1 | 5 | 0.43333 | 0.70565 | 0.3900 | **0.00010** |
|  | AG2 | 11 | 0.50000 | 0.62203 | 0.1989 | **0.02529** |
|  | AG5 | 6 | 0.63333 | 0.73785 | 0.1437 | **0.02147** |
|  | AC5 | 3 | 0.20000 | 0.45028 | 0.5601 | **0.00000** |
|  | AG1 | 4 | 0.13333 | 0.41695 | 0.6839 | **0.00000** |
|  | A10 | 3 | 0.63333 | 0.61525 | 0.0299 | **0.00043** |
|  | B07 | 7 | 0.40000 | 0.71525 | 0.4450 | **0.00000** |
|  | AC7 | 7 | 0.16667 | 0.70169 | 0.7656 | **0.00000** |
|  |  |  |  |  |  |  |
| INT-4 | AT1 | 10 | 0.66667 | 0.81864 | 0.1882 | **0.00000** |
|  | AG7 | 7 | 0.73333 | 0.68531 | 0.0714 | 0.24051 |
|  | AC1 | 4 | 0.30000 | 0.58927 | 0.4952 | **0.00001** |
|  | AG2 | 13 | 0.80000 | 0.91751 | 0.1300 | **0.00000** |
|  | AG5 | 6 | 0.43333 | 0.75537 | 0.4305 | **0.00000** |
|  | AC5 | 5 | 0.20000 | 0.63785 | 0.6901 | **0.00000** |
|  | AG1 | 5 | 0.26667 | 0.70960 | 0.6282 | **0.00000** |
|  | A10 | 3 | 0.40000 | 0.54407 | 0.2681 | 0.10578 |
|  | B07 | 7 | 0.73333 | 0.71582 | 0.0249 | **0.00151** |
|  | AC7 | 4 | 0.03333 | 0.27062 | 0.8787 | **0.00000** |
|  |  |  |  |  |  |  |
| URB-1 | AT1 | 9 | 0.56667 | 0.76328 | 0.2609 | **0.00000** |
|  | AG7 | 3 | 0.00000 | 0.41356 | 1 | **0.00000** |
|  | AC1 | 7 | 0.30000 | 0.68305 | 0.5650 | **0.00000** |
|  | AG2 | 10 | 0.76667 | 0.84407 | 0.0931 | 0.22520 |
|  | AG5 | 6 | 0.36667 | 0.76836 | 0.5271 | **0.00000** |
|  | AC5 | 4 | 0.33333 | 0.54915 | 0.3971 | **0.00117** |
|  | AG1 | 4 | 0.53333 | 0.68192 | 0.2208 | **0.00000** |
|  | A10 | 5 | 0.50000 | 0.58870 | 0.1529 | **0.02051** |
|  | B07 | 7 | 0.46667 | 0.54068 | 0.1389 | 0.09094 |
|  | AC7 | 7 | 0.23333 | 0.47684 | 0.5149 | **0.00000** |
|  |  |  |  |  |  |  |
| URB-2 | AT1 | 10 | 0.46667 | 0.84463 | 0.4517 | **0.00000** |
|  | AG7 | 7 | 0.86667 | 0.76610 | 0.1338 | **0.00077** |
|  | AC1 | 4 | 0.23333 | 0.57119 | 0.5956 | **0.00000** |
|  | AG2 | 12 | 0.93333 | 0.84802 | 0.1025 | 0.13499 |
|  | AG5 | 8 | 0.76667 | 0.83390 | 0.0819 | **0.00332** |
|  | AC5 | 8 | 0.40000 | 0.68475 | 0.4200 | **0.00000** |
|  | AG1 | 4 | 0.06667 | 0.71356 | 0.9080 | **0.00000** |
|  | A10 | 5 | 0.43333 | 0.66384 | 0.3511 | **0.01421** |
|  | B07 | 6 | 0.20000 | 0.48870 | 0.5949 | **0.00000** |
|  | AC7 | 9 | 0.70000 | 0.71525 | 0.0217 | **0.00000** |

Significant *P*-values in bold.
